# Supplementary material for: Aberrant HLA-DR expression in the conjunctival epithelium after autologous serum treatment in patients with graft-versus-host disease or Sjögren’s syndrome
Source: PLoS One. 2020 Apr 21;15(4):e0231473. doi: 10.1371/journal.pone.0231473 (PMC7173771; doi:10.1371/journal.pone.0231473)
Supplement: S1 Data — (PDF) [file pone.0231473.s001.pdf]

cells/per field											%				
all			GVHD												
			epithelial				LC				epithelial				
patient			before	na mm2	after	na mm2	before	na mm2	after	na mm2	before	after	patient		
P1	241	OP	12,0	89,2	16,3	121,2	0,1	0,7	0,3	2,2	47	58	P13	225	OP
		OL	14,2	105,6	16,7	124,2	0,0	0,0	0,1	0,7	94	93			OL
P2	219	OP	36,0	267,5	91,3	678,7	5,1	37,7	10,8	80,1	18	24	P14	226	OP
		OL	0,5	3,7	102,3	760,6	0,2	1,5	6,5	48,3	1	46			OL
P3	219	OPlbt	1,3	9,9	64,2	477,3	0,0	0,0	0,6	4,5	3	31	P15	200	OP
		OLlbt	7,7	57,2	179,0	1330,9	0,9	6,7	6,6	49,1	17	70			OL
P4	231	OP	29,9	222,3	32,1	238,7	0,1	0,7	0,0	0,0	53	84	P16	220	OP
		OL	8,6	63,9	14,2	105,6	0,0	0,0	0,0	0,0	10	13			OL
P7	230	OP	70,7	525,7	38,5	286,2	2,1	15,6	0,8	5,9	42	45	P17	240	OP
		OL	24,9	184,8	46,2	343,5	0,6	4,5	1,9	14,1	32	45			OL
P6	222	OP	57,3	425,9	56,1	417,4	0,5	3,5	0,6	4,2	28	56	P18	218	OP
		OL	13,2	98,1	11,9	88,5	0,7	5,2	0,0	0,0					OL
P8	250	OP	2,4	17,8	22,9	170,3	0,4	3,0	0,8	5,9	3	21	P19	246	OP
		OL	1,8	13,4	3,6	26,8	1,1	8,2	1,8	13,4	2	0			OL
P5	235	OP	21,6	160,6	11,6	86,2	0,0	0,0	0,5	3,7	88	83	P20	247	OP
P9	253	OP	20,4	151,7	155,4	1155,4	0,6	4,5	2,4	17,8	8	38			OL
		OL	109,8	816,4	48,2	358,4	0,0	0,0	0,6	4,5	53	39	P21	248	OP
P10	238	OP	0,1	0,7	4,5	33,5	0,0	0,0	0,0	0,0	0	4			OL
		OL	1,3	9,7	1,8	13,4	0,1	0,7	0,2	1,5	2	4	P22	242	OP
P11	253	OP	0,1	0,7	4,6	34,2	2,1	15,6	4,5	33,5	29	49			OL
		OL	24,7	183,6	23,8	177,0	1,7	12,6	1,6	11,9	46	54	P		OP
P12	223	OP	5,8	43,1	3,5	26,0	0,5	3,7	1,0	7,4	10	7			OL
		OL	1,6	11,9	0,7	5,2	1,3	9,7	1,1	8,2	8	5	P24		OP
suma			465,8	3463,5	949,4	7058,9	18,1	134,2	42,6	317,0	594,0	869,0			OL
prumer			20,3	150,6	41,3	306,9	0,8	5,8	1,9	13,8	27,0	39,5			
SD			26,9	199,9	48,7	362,2	1,1	8,5	2,7	20,2	27,5	27,9			

prim SS

epithelial				LC				epithelial	
before		after		after		after		before	after
0,3	2,2	0,0	0,0	0,2	1,5	0,0	0,0	0,3	0,0
0,1	0,7	0,1	0,7	0,4	3,0	1,1	8,2	0,1	0,1
9,3	69,1	6,5	48,3	0,1	0,7	0,0	0,0	33,0	33,0
7,1	52,8	1,5	11,2	0,0	0,0	0,0	0,0	51,0	27,0
55,4	411,9	43,9	326,4	0,0	0,0	0,0	0,0	81,0	71,0
5,4	40,1	0,4	3,0	0,0	0,0	0,4	3,0	26,0	6,0
148,2	1101,9	27,2	202,2	0,8	5,9	0,1	0,7	64,0	2,0
0,2	1,5	0,8	5,9	0,0	0,0	0,0	0,0	5,0	5,0
7,3	54,3	2,1	15,6	0,3	2,2	0,3	2,2	15,0	0,0
16,7	124,2	3,9	29,0	0,4	3,0	0,6	4,5	4,0	5,0
274,0	2037,2	235,0	1747,2	4,7	34,9	5,3	39,4	72,0	68,0
303,0	2252,8	0,7	5,2	2,5	18,6	0,7	5,2	40,0	1,0
92,7	689,2	108,4	805,9	0,0	0,0	1,8	13,4	80,0	76,0
2,0	14,9	47,6	353,9	0,0	0,0	0,0	0,0	54,0	90,0
74,8	556,1	62,1	461,7	3,4	25,3	1,5	11,2	26,0	26,0
4,4	32,7	24,6	182,9	0,1	0,7	1,0	7,4	4,0	17,0
0,7	5,2	2,0	14,9	0,5	3,7	0,3	2,2	0,0	2,0
0,6	4,5	0,3	2,2	0,4	3,0	0,1	0,7	0,0	0,0
27,4	203,7	239,5	1780,7	1,3	9,7	3,5	26,0	31,0	76,0
4,7	34,9	21,6	160,6	0,1	0,7	0,0	0,0	4,0	11,0
30,4	226,0	40,3	299,6	0,6	4,5	2,5	18,6	16,0	17,0
63,3	470,6	36,4	270,6	1,9	14,1	4,5	33,5	21,0	10,0
0,9	6,7	2,6	19,3	1,1	8,2	0,5	3,7	0,0	0,0
0,0	0,0	0,0	0,0	0,7	5,2	0,5	3,7	0,0	0,0
1128,9	8393,3	907,5	6747,2	19,5	145,0	24,7	183,6	627,4	543,1
47,0	349,7	37,8	281,1	0,8	6,0	1,0	7,7	26,1	22,6
83,2	618,6	66,9	497,3	1,2	8,9	1,5	11,1	27,5	29,8

density

epi before	after	LC before	LC after
89,2	121,2	0,7	2,2
105,6	124,2	0,0	0,7
267,5	678,7	37,7	80,1
3,7	760,6	1,5	48,3
9,9	477,3	0,0	4,5
57,2	1330,9	6,7	49,1
222,3	238,7	0,7	0,0
63,9	105,6	0,0	0,0
525,7	286,2	15,6	5,9
184,8	343,5	4,5	14,1
425,9	417,4	3,5	4,2
98,1	88,5	5,2	0,0
17,8	170,3	3,0	5,9
13,4	26,8	8,2	13,4
160,6	86,2	0,0	3,7
151,7	1155,4	4,5	17,8
816,4	358,4	0,0	4,5
0,7	33,5	0,0	0,0
9,7	13,4	0,7	1,5
0,7	34,2	15,6	33,5
183,6	177,0	12,6	11,9
43,1	26,0	3,7	7,4
11,9	5,2	9,7	8,2
2,2	0,0	1,5	0,0
0,7	0,7	3,0	8,2
69,1	48,3	0,7	0,0
52,8	11,2	0,0	0,0
411,9	326,4	0,0	0,0
40,1	3,0	0,0	3,0
1101,9	202,2	5,9	0,7

1,5	5,9	0,0	0,0
54,3	15,6	2,2	2,2
124,2	29,0	3,0	4,5
2037,2	1747,2	34,9	39,4
2252,8	5,2	18,6	5,2
689,2	805,9	0,0	13,4
14,9	353,9	0,0	0,0
556,1	461,7	25,3	11,2
32,7	182,9	0,7	7,4
5,2	14,9	3,7	2,2
4,5	2,2	3,0	0,7
203,7	1780,7	9,7	26,0
34,9	160,6	0,7	0,0
226,0	299,6	4,5	18,6
470,6	270,6	14,1	33,5
6,7	19,3	8,2	3,7
0,0	0,0	5,2	3,7
252,3	293,7	5,9	10,7
464,6	427,3	8,5	16,2

% all epi

47	58
94	93
18	24
1	46
3	31
17	70
53	84
10	13
42	45
32	45
28	56
3	21
2	0
88	83
8	38
53	39
0	4
2	4
29	49
46	54
10	7
8	5
0,3	0,0
0,1	0,1
33,0	33,0
51,0	27,0
81,0	71,0
26,0	6,0
64,0	2,0

5,0	5,0
15,0	0,0
4,0	5,0
72,0	68,0
40,0	1,0
80,0	76,0
54,0	90,0
26,0	26,0
4,0	17,0
0,0	2,0
0,0	0,0
31,0	76,0
4,0	11,0
16,0	17,0
21,0	10,0
0,0	0,0
0,0	0,0
26,6	30,7
