# Supplementary material for: Aberrant HLA-DR expression in the conjunctival epithelium after autologous serum treatment in patients with graft-versus-host disease or Sjögren’s syndrome
Source: PLoS One. 2020 Apr 21;15(4):e0231473. doi: 10.1371/journal.pone.0231473 (PMC7173771; doi:10.1371/journal.pone.0231473)
Supplement: S2 Data — (PDF) [file pone.0231473.s002.pdf]

age gvhd	age SS
42	41
49	59
50	37
37	34
49	31
25	40
43	65
56	52
64	
	47
47	
	71
65	
	53
62	
	64
49,08333	48,18182
p(T.test)	0,935678

man	pred	po	narust o %
P1	**89,2**	**121,2**	35,83333
	**105,6**	**124,2**	17,60563
P2	**267,5**	**678,7**	153,742
	**3,7**	**760,6**	nezahrnutc
P3	**9,9**	**477,3**	4727,068
	**57,2**	**1330,9**	2224,675
P4	**222,3**	**238,7**	7,35786
	**63,9**	**105,6**	65,11628
P5	**160,6**	**86,2**	-46,2963
P6	**425,9**	**417,4**	-1,99501
	**98,1**	**88,5**	-9,84848
		**prumer**	**717,3258**
		**p (T.test)**	**0,829115**
suma	**3463,5**	**7058,9**	103,8092
range	**150,6**	**306,9**	103,8092
SD	**199,9**	**362,2**	81,22294

women	pred	po	narust o %
P7	**525,7**	**286,2**	-45,5446
	**184,8**	**343,5**	85,91549
P8	**17,8**	**170,3**	854,1667
	**13,4**	**26,8**	100
P9	**151,7**	**1155,4**	661,7647
	**816,4**	**358,4**	-56,102
P10	**0,7**	**33,5**	4400
	**9,7**	**13,4**	38,46154
P11	**0,7**	**34,2**	4500
	**183,6**	**177,0**	-3,64372
P12	**43,1**	**26,0**	-39,6552
	**11,9**	**5,2**	-56,25
			**869,9261**
