# Supplementary material for: Aberrant HLA-DR expression in the conjunctival epithelium after autologous serum treatment in patients with graft-versus-host disease or Sjögren’s syndrome
Source: PLoS One. 2020 Apr 21;15(4):e0231473. doi: 10.1371/journal.pone.0231473 (PMC7173771; doi:10.1371/journal.pone.0231473)
Supplement: Attachment — Submitted filename: Response to Reviewers.docx [file pone.0231473.s003.docx]

February 4, 2020

Alexander V. Ljubimov, Ph.D.

Academic Editor

PLOS ONE

Dear Dr. Ljubimov,

We thank the reviewers for their helpful comments, and we have addressed the comments from the referees as follows:

PONE-D-19-27094

Response to Reviewers

**1**

**The authors performed an observational study of subjects with dry eye either secondary to o GVHD or primary Sjogren's syndrome to correlate clinical symptoms with impression cytology of the conjunctiva and to determine the number of HLADR+ cells before and after treatment with autologous serum eye drops. The reviewers found some problems with the paper that the authors need to address.**

**It is noted that there are many confounders in study - sex, previous treatment, reliability of counting + HLADR cells from IC stained filters. These need to be evaluated.**

Potential confounder factors has been analyzed where possible. No statistical difference was found in the age of patients with GVHD and SS respectively (p=0,94). See Statistical analysis and Results sections in the manuscript.

Due to the fact that group of SS involves just females, and GVHD patients included both males and females, statistical difference of HLA-Dr positivity was calculated separately for GVHD males and GVHD females. No significant difference was found. This information was included in the manuscript.

The paragraph regarding treatment of patients (Material and Methods section) was rewritten separately for GVHD and SS patients respectively ((“Sixteen patients were under the treatment with preservative-free artificial tear eye drops (nine from GVHD and seven from SS group respectively), and eight patients with artificial tears containing oxychloro complex (Purite) or Polyquad preservatives (four patients per each group). Seven patients, all from the GvHD group, were on systemic immunosuppressive therapy and antiviral therapy. In the SS group, two patients were receiving systemic corticosteroids.”)). The therapy of dry eye disease is highly individualized, the broad spectrum of medical treatment does not allow statistical analysis. It should be, however, noted that therapy regimen was not changed for any patient involved in the study for at least two months prior to the application of AS and during the follow-up period, which we consider to be an important factor.

The sentence ”The HLA-DR-positive cells were clearly discernible from negative cells.” was added to Material and Methods section.

**2. It is not clear if the readers of IC were " blinded" to pre vs. post AS treatment when evaluating samples.**

Images were evaluated by two independent investigators blinded to the experimental conditions (patient identity, before and after AS specimens). Following sentence was added to the text.

“The density of HLA-DR-positive epithelial cells and Langerhans cells was assessed independently by two researchers (KJ, VV) blinded to the experimental conditions using a NIS Elements image analysis system (Laboratory Imaging, Prague, Czech Republic).”

Yours sincerely, Katerina Jirsova

Assoc. Prof. Katerina Jirsova, Ph.D.

Laboratory of the Biology and Pathology of the Eye

Institute of Biology and Medical Genetics, First Faculty of Medicine, Charles University and General University Hospital in Prague,

Albertov 4, 128 00 Prague 2,

Czech Republic

Tel: 00420 224 968 006, E-mail: katerina.jirsova@lf1.cuni.cz
